# Germination and Early Establishment Requirements of *Salicornia europaea* aggr. as a Candidate Edible Crop for Saline Environments

**DOI:** 10.3390/plants15060920

**Published:** 2026-03-16

**Authors:** Konstantinos Koularmanis, Maria Androudi, Katerina Papanastasi, Eleni Maloupa, Athanasios Koukounaras, Vassilis Aschonitis, Katerina Grigoriadou

**Affiliations:** 1 Institute of Plant Breeding and Genetic Resources, Hellenic Agricultural Organization—DIMITRA, P.O. Box 60458, 57001 Thessaloniki, Greece; kkoularmanis@elgo.gr (K.K.); mandroudi@elgo.gr (M.A.); kpapanastasi@elgo.gr (K.P.); emaloupa@elgo.gr (E.M.); 2 Laboratory of Vegetable Crops, School of Agriculture, Aristotle University of Thessaloniki, 54124 Thessaloniki, Greece; thankou@agro.auth.gr; 3 Soil & Water Resources Institute, Hellenic Agricultural Organization—DIMITRA, 57001 Thessaloniki, Greece; v.aschonitis@elgo.gr

**Keywords:** gibberellic acid, light intensity, propagation, salinity, seedling

## Abstract

Halophyte plants constitute vital resources for the advancement of sustainable agricultural practices in soils affected by salinity; however, the precise germination requirements for these species are still inadequately investigated. In this study, we examined how *Salicornia europaea* aggr., a succulent edible halophyte species, germinates under different genetic, environmental, and hormonal conditions such as gibberellic acid (GA_3_), testing the effects of genotype, light exposure, and salinity stress on seed and early seedling development. Two genotypes (GR-1-BBKK-24.6196 and GR-1-BBKK-25.6212) were examined across a range of GA_3_ (0, 250, 500 ppm), light intensity (40 and 80 μmol m^−2^ s^−1^), and salt concentrations (0 and 1% NaCl). At the seedling stage, four NaCl concentrations were used (0, 50, 100, 200 mM NaCl). Our data showed that *S. europaea* seeds do not exhibit dormancy—GA_3_ treatment had no effect on germination success. Dark conditions and salt exposure both hindered germination, whereas the highest light intensity (80 μmol m^−2^ s^−1^) improved it considerably. Salt stress progressively slowed seedling growth. Seedling development was enhanced by 200 mM NaCl demonstrating inconsistency of the effect of salinity between the seed and seedling stages. Overall, our work demonstrates that the germination in *S. europaea* varies substantially between genotypes, with sufficient light and low salt being particularly important for maximizing seed germination, while 200 mM NaCl seems to promote seedling growth.

## 1. Introduction

Soil salinization poses a critical threat to global food security, impacting around 20% of irrigated land and increasing by 1.5 million hectares yearly, necessitating the investigation of halophyte plants that can thrive in high salinity and contribute to sustainable agricultural practices and ecosystem benefits [1,2]. Given the well-documented taxonomic complexity of the *Salicornia* group, the plant material is referred to as *Salicornia europaea* aggr., following the aggregate concept. This framework acknowledges the presence of micro-taxa within the group while focusing on the selected genotypes [3]. *Salicornia europaea* aggr., or the common glasswort, is a halophyte prevalent in temperate coastal regions, known for its exceptional salt tolerance and potential as a crop due to its edible parts and biomass productivity, which necessitates in-depth research into its germination dynamics and the interplay of genetic and environmental factors for effective cultivation [3]. Seed germination in halophytes is described as an intricate physiological process that balances between successful establishment under zero or low-saline while the challenges posed by hypersaline surrounding conditions may lead to a reduction from 75 to 100% to less than 10% in germination (%) [4]. Besides salinity tolerance or intolerance lies the complex seed heteromorphism of many halophytes, recognized as a basic adaptation strategy that allows plants to cope with variable and unpredictable environments [5,6]. In many cases, seeds exhibit dormancy and, to alleviate this, researchers employ supplementary gibberellic acid (GA_3_) applications in addition to the natural levels in seeds, which is considered as the most efficient dormancy-breaking method [7,8,9].

The literature highlights key factors affecting halophyte germination, such as salinity, light, temperature, and hormonal influences, with evidence indicating that *Salicornia rubra* exhibits a dose-dependent response to NaCl, revealing both reductions and paradoxical enhancements in germination under varying salinity levels [10]. Concerning diminished salinity conditions, it has been proven that the use of fresh water over saline significantly improved the germination of halophytes in a greenhouse environment grown under various substrates [11]. As for increased salinity, salinity levels not exceeding 50 mM NaCl did not adversely affect germination rates of selected halophyte species [12]. The seeds of halophytes possess the ability to recuperate from saline shock and initiate the germination process once the salinity levels are diminished, a phenomenon that may occur posterior to precipitation events. In certain instances, the process of imbibition in a low-salinity solution may facilitate osmo-priming, thereby enhancing the germination rate [4,13]. Concurrently, salinity may exert a synergistic effect alongside light [14]. The light requirements for the germination of halophytes are not clearly defined yet, in contrast to glycophytic species [15]. Additional research on halophyte seed germination has shown that light serves as a crucial component, often resulting in significantly higher germination percentages compared to conditions of total darkness [13,16,17].

Although there is existing documentation regarding the influence of light on the development of *S. europaea* [15], the specific light intensity thresholds necessary for optimal germination have not been thoroughly examined. This deficiency in knowledge is particularly crucial, considering that the availability of light in the natural habitats of halophytes fluctuates significantly due to tidal patterns, seasonal variations, and the heterogeneity of microhabitats [18]. Genetic variability within halophyte species constitutes a vital yet underutilized asset for comprehending adaptive strategies and formulating enhanced propagation methodologies. Recent research has demonstrated considerable differences in salt tolerance among different genotypes of *S. europaea*, influenced by their natural habitat, wherein the local environmental context plays a significant role in shaping stress response characteristics [19]. Nonetheless, there is a paucity of controlled investigations that address genotype–environment interactions during the germination process, despite their critical relevance for both theoretical insights and practical implementations.

Beyond the genotypic and environmental parameters influencing germination, characterizing the subsequent seedling phase is essential for understanding the early-life species response to salinity. Specifically, although the favorable effect of salinity on juvenile *S. europaea* plants starting from 200 and gradually declining at 800 mM NaCl has been previously studied in the literature [20], the range of desirable salinity levels at the seedling stage, along with genotypic interference, has not been comprehensively investigated to date.

The objective of this study was to assess the cultivation framework of *S. europaea* by identifying its reproductive boundaries and discovering how its environmental plasticity is influenced by genotypic variation during the seed and early seedling phase. Accordingly, we hypothesized that exogenous GA_3_ application might be required to alleviate dormancy constraints. We further hypothesized that the absence or minimal salinity levels would favor the initial germination phase, while moderate light intensities would maximize germination percentage. Regarding the early seedling phase, our hypothesis was based on high genotypic interaction and maximum seedling growth at the highest tested salinity (200 mM NaCl), as existing reports indicate that the favorable effects of salinity on juvenile *S. europaea* begin at 200 mM NaCl [20,21]. By integrating these stages, this research aims to propose a comprehensive sexual propagation protocol—spanning from successful in vitro germination to robust seedling development—providing a scientific basis for practical applications such as direct sowing in degraded soils.

## 2. Results

### 2.1. GA_3_ Seed Dormancy In Vitro Test

No dormancy effect was noted between genotypes, and this was evidenced by the absence of statistically significant differences between control (0 GA_3_) and both levels of GA_3_ treatment (250, 500 GA_3_). Specifically, GR-1-BBKK-24.6196 showed 50.66% germination while GR-1-BBKK-25.6212 reached 50.76% (*p* > 0.05). Germination speed followed the same pattern, showing values of 6.22 and 6.80% (for GR-1-BBKK-24.6196 and GR-1-BBKK-25.6212, respectively) as presented in Table 1. Regarding the interaction between the genotype and the GA_3_ level, no statistically significant effect was observed in any of the parameters measured (Table 1). In contrast, statistically significant differences were observed both in terms of the effect of genotype on seedling length (0.77 to 0.61, *p* < 0.05) and the effect of GA_3_ level on the Vigor I growth index, with the 500 GA_3_ level differentiating from the 0 GA_3_ level (48.61 to 25.09, *p* < 0.05). Overall, the parameters measured did not indicate any dependence of the seeds on dormancy.

### 2.2. Synergistic 3-Way Effects on the Germination Kinetics of S. europaea aggr.

Regarding the three-way analysis of *S. europaea* seed germination, all three factors exerted significant effects on the parameters measured (Table 2). The significance of η_p_^2^ reveal a strong biological relevance, suggesting that these three factors synergistically affect germination success of *S. europaea* aggr. Specifically, germination (%) was influenced by the simple main effects of genotype, light intensity and salinity with salinity explaining the highest percentage of variance based on η_p_^2^ index (η_p_^2^ = 60, Table 2). Regarding interactions effect the interaction between light intensity × salinity presented significant differences but explaining lower percentage of the overall variance compared to simple main effects (η_p_^2^ = 16.1, Table 2).

Regarding the germination speed parameter, all three main effects of the factors studied herein showed a statistically significant effect, and their interaction was also significant but with a smaller contribution to the total variability (Table 2, η_p_^2^ = 12.7). Proceeding with the study of the first of the two parameters for evaluating seedling growth, Vigor I index was influenced by the simple main effects of genotype, light intensity, and salinity, along with the interactions of G × LI, G × S and LI × S. Among the statistical effects, LI and salinity simple main effects contributed the most to the overall variance (Table 2, η_p_^2^ = 61.4 and 41.1, respectively). Finally, all simple main effects of G, LI, S and LI × S interaction effect opposed significant effect on Vigor II index. Specifically, LI and S explained the highest percentage of variance in terms of η^2^ effect size index (68.5 and 69.9, respectively).

**Figure 1:**
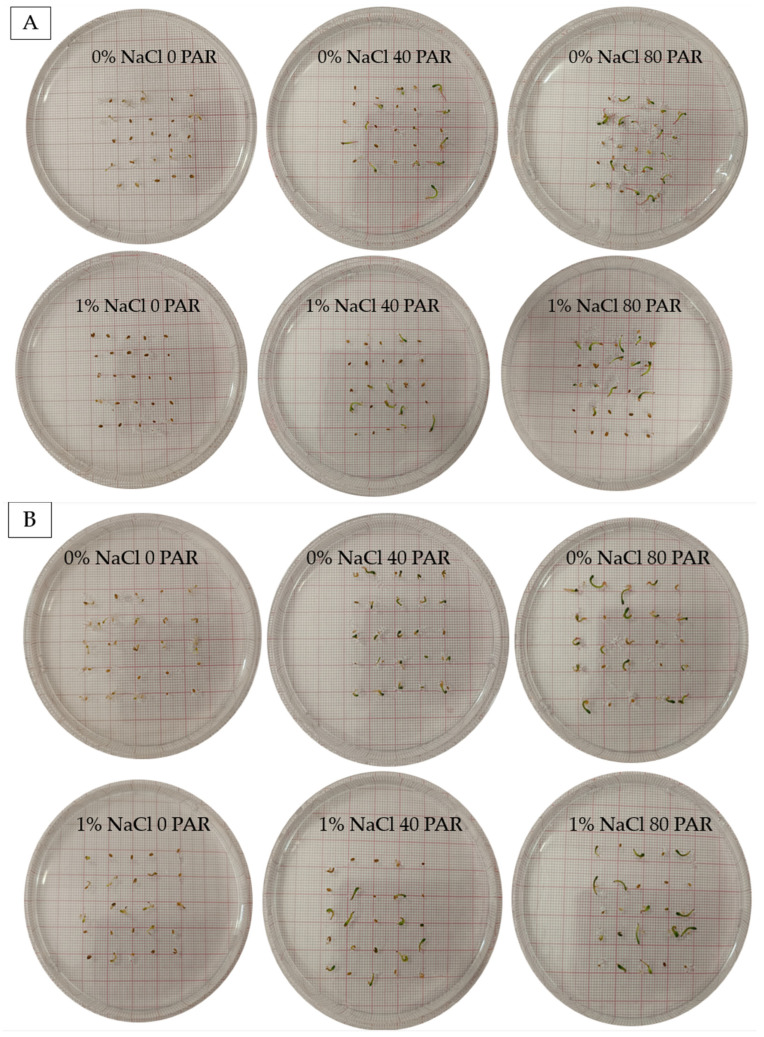
Comparative performance images of germination of *S. europaea* seeds treated with 0 NaCl-0 PAR, 0 NaCl-40 PAR, 0 NaCl-80 PAR and 1% NaCl-0 PAR, 1% NaCl-40 PAR, 1% NaCl-80 PAR for GR-1-BBKK-25.6212 (**A**) and GR-1-BBKK-24.6196 (**B**) genotypes, respectively (after 20 days).

**Figure 2:**
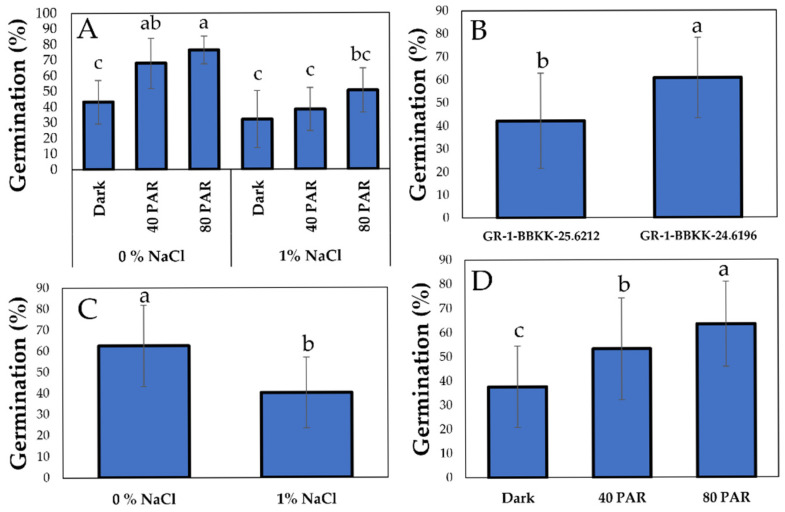
Combined effect of salinity (0, 1% NaCl) × light intensity (0, 40 and 80 µmol m^−2^ s^−1^) interaction on germination (%) of *S. europaea* aggr. (**A**). Effect of genotype (GR-1-BBKK-25.6212 and GR-1-BBKK-24.6196) on germination (%) of *S. europaea* aggr. (**B**). Effect of salinity (0, 1% NaCl) on germination (%) of *S. europaea* aggr. (**C**). Effect of light intensity (0, 40 and 80 µmol m^−2^ s^−1^) on germination (%) of *S. europaea* aggr. (**D**). Data shown are the mean values (*n* = 5 ± SD). Different letters between each bar indicate the significance of the difference (*p* < 0.05) based on Tukey’s HSD post hoc test and Student’s *t*-test.

Seed germination was totally enhanced by the absence of salinity in the growing medium, along with both light treatments (Figure 1). Specifically, regarding the interaction of factors, statistically significant differences appeared both between the highest light intensity treatment (80 LI) in 0% NaCl substrate and all treatments of 1% NaCl, as well as between the two highest light intensity treatments (80 LI) both in saline and non-saline substrates (Figure 2A, *p* < 0.05). Regarding the two genotypes studied, significant differences appeared, once again highlighting the effect of genotype on germination (Figure 2B, *p* < 0.05). Additionally, salinity exerted a statistically significant impact, with the absence of NaCl in the culture substrate yielding notably higher averages than its presence (Figure 2C, *p* < 0.05). At the same time, the LI factor was also significant, as the highest light intensity level (80 PAR) differed from both 40 PAR and 0 PAR, respectively (Figure 2D, *p* < 0.05).

**Figure 3:**
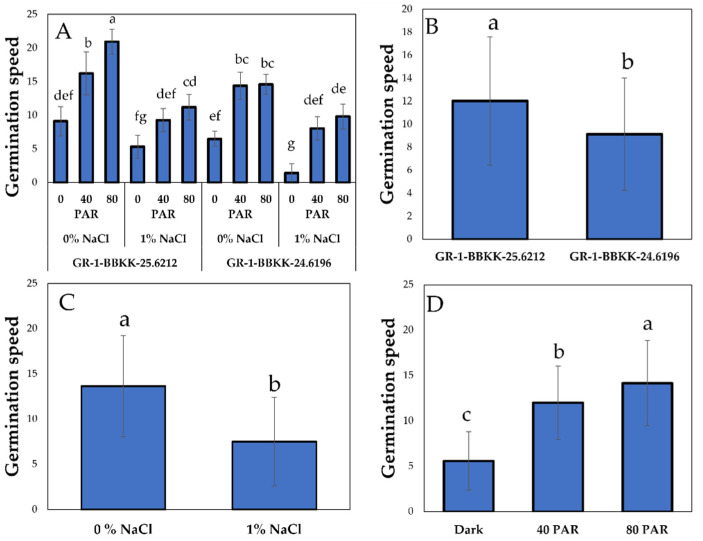
Combined effect of genotype (GR-1-BBKK-25.6212 and GR-1-BBKK-24.6196) × salinity (0, 1% NaCl) × light intensity (0, 40 and 80 µmol m^−2^ s^−1^) interaction on germination speed index of *S. europaea* aggr (**A**). Effect of genotype (GR-1-BBKK-25.6212 and GR-1-BBKK-24.6196) on germination speed index of *S. europaea* aggr (**B**). Effect of salinity (0, 1% NaCl) on germination speed index of *S. europaea* aggr. (**C**). Effect of light intensity (0, 40 and 80 µmol m^−2^ s^−1^) on germination speed index of *S. europaea* aggr. (**D**). Data shown are the mean values (*n* = 5 ± SD). Different letters between each bar indicate the significance of the difference (*p* < 0.05) based on Tukey’s HSD post hoc test and Student’s *t*-test.

Regarding the analysis of variability for germination speed, the analysis showed the effect of a three-way (Genotype × Light intensity × Salinity). GR-1-BBKK-25.6212 genotype under no salinity treatment and the highest LI (80 PAR) exhibited the highest germination speed compared to any other combined effect treatment. At the same time, dark condition (0 PAR) treatments resulted in the lowest germination rates, regardless of the salinity levels in the growth medium (Figure 3A, *p* < 0.05). Genotype-wise, a contrasting pattern was observed in comparison with the germination percentage, and GR-1-BBKK-25.6212 showed a statistically significantly higher germination rate (Figure 3B, *p* < 0.05). Following that, simple main effects of salinity and LI showed the same trend as germination (%), resulting in higher germination speed under 0 NaCl treatment and elevated LI (Figure 3C,D, *p* < 0.05).

**Figure 4:**
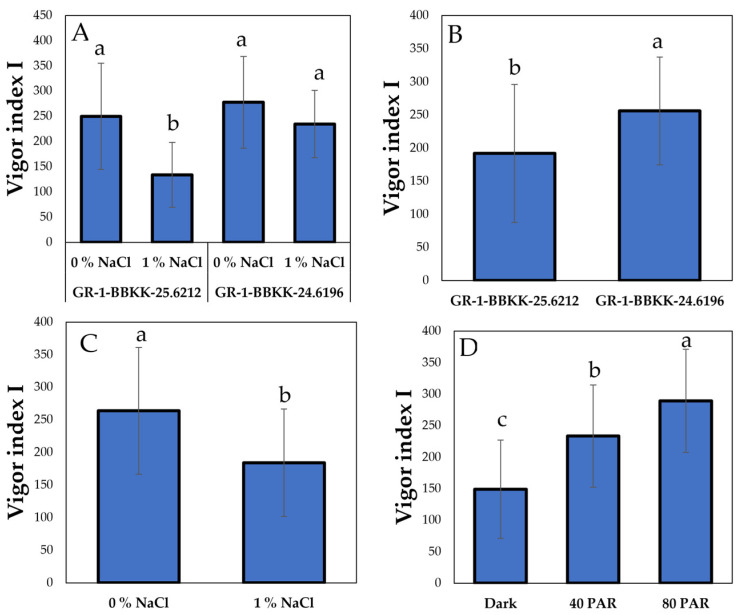
Combined effect of genotype (GR-1-BBKK-25.6212 and GR-1-BBKK-24.6196) × salinity (0, 1% NaCl) interaction on Vigor Index I of *S. europaea* aggr. (**A**). Effect of genotype (GR-1-BBKK-25.6212 and GR-1-BBKK-24.6196) on Vigor Index I of *S. europaea* aggr. (**B**). Effect of salinity (0, 1% NaCl) on Vigor Index I of *S. europaea* aggr. (**C**). Effect of light intensity (0, 40 and 80 µmol m^−2^ s^−1^) on Vigor Index I of *S. europaea* aggr. (**D**). Data shown are the mean values (*n* = 5 ± SD). Different letters between each bar indicate the significance of the difference (*p* < 0.05) based on Tukey’s HSD post hoc test and Student’s *t*-test.

Vigor indexes representing the growth of the seedlings were also affected by the interaction and simple main effects of the factors studied herein. Specifically, regarding the interaction between genotype and salinity, the 1% salinity level of GR-1-BBKK-25.6212 genotype showed significantly lower growth compared to the other treatments (Figure 4A, *p* < 0.05). In addition, GR-1-BBKK-24.6196 genotype surpassed GR-1-BBKK-25.6212 genotype in terms of Vigor Index I (Figure 4B, *p* < 0.05). The salinity factor exhibited an analogous effect in Vigor Index I as previously measured parameters, with the 0% NaCl treatment differing statistically significantly from the 1% NaCl treatment (Figure 4C, *p* < 0.05). Once more, the LI factor showed a statistically significant effect as higher LI (80 and 40 PAR, respectively), presenting elevated average values in Vigor Index I compared with 0 PAR (Figure 4D, *p* < 0.05).

**Figure 5:**
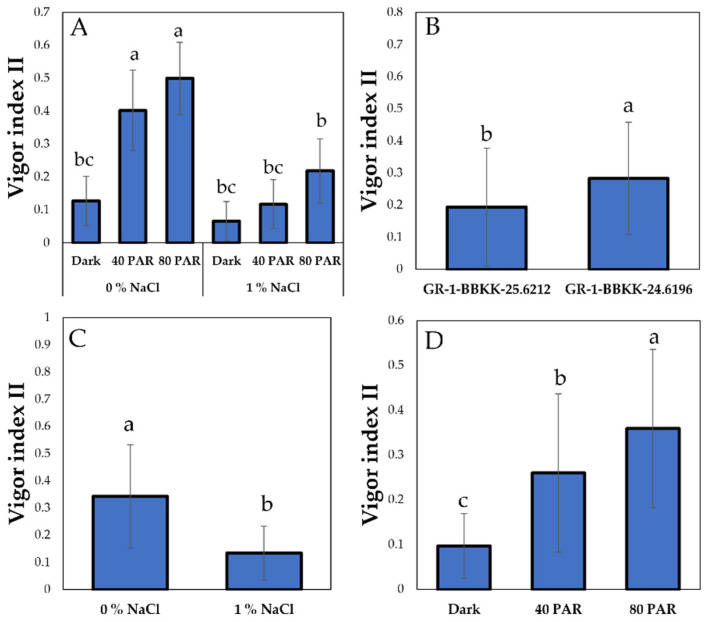
Combined effect of salinity (0, 1% NaCl) × light intensity (0, 40 and 80 µmol m^−2^ s^−1^) interaction on Vigor Index II of *S. europaea* aggr. (**A**). Effect of genotype (GR-1-BBKK-25.6212 and GR-1-BBKK-24.6196) on Vigor Index II of *S. europaea* aggr. (**B**). Effect of salinity (0, 1% NaCl) on Vigor Index II of *S. europaea* aggr. (**C**). Effect of light intensity (0, 40 and 80 µmol m^−2^ s^−1^) on Vigor Index II of *S. europaea* aggr. (**D**). Data shown are the mean values (*n* = 5 ± SD). Different letters between each bar indicate the significance of the difference (*p* < 0.05) based on Tukey’s HSD post hoc test and Student’s *t*-test.

Vigor Index II was also affected by the light intensity × salinity interaction effect along with genotype, salinity and LI simple main effects. Clear differences were observed between the two light intensity levels (40, 80 PAR) under conditions of 0% NaCl and all other treatments (Figure 5A, *p* < 0.05). Furthermore, GR-1-BBKK-24.6196 showed an enhanced Vigor Index II compared to GR-1-BBKK-25.6212 (Figure 5B, *p* < 0.05). Regarding the main effect of salinity, the absence of salinity in the growing substrate resulted in more robust seedlings in terms of Vigor Index II (Figure 5D, *p* < 0.05). At the same time, increased light intensity levels exhibited an enhancing effect on Vigor Index II (Figure 5D, *p* < 0.05).

### 2.3. Salinity and Genotype Effect of In Vitro Seedlings

Analysis of variance for seedling length was assessed both within each measured day after start of the experiment (DA) and between each DA. Starting at 7 DA, only the G × S interaction had a significant effect in seedling length. Specifically, 50 mM NaCl treatment of GR-1-BBKK-24.6196 and 100 mM NaCl treatment of GR-1-BBKK-25.6212 genotypes showed significant differences among other treatments tested (Table 3, *p* < 0.05). At 15 DA, the same pattern was noted as 0 mM NaCl treatment differed significantly from 100 and 200 mM NaCl under the same genotype (GR-1-BBKK-25.6212). Regarding the last two DAs (40 and 60, respectively), the genotype exhibited a significant effect on seedling length, with GR-1-BBKK-25.6212 showing increased average values compared to GR-1-BBKK-24.6196 both at 40 DA (2.11 cm to 1.59 cm, respectively) and at 60 DA (2.33 cm to 1.60 cm, respectively). In particular, at 60 DA, the salinity effect resulted in an enhanced seedling length between 0 and 200 mM NaCl (Table 3, *p* < 0.05). Concerning fresh weight as measured at 60 DA, it appeared to be genotype-dependent, with GR-1-BBKK-25.6212 presenting significantly higher (*p* < 0.05) average values than GR-1-BBKK-24.6196 (0.045 to 0.016, respectively). At the same time, no significant differences were observed in terms of dry weight. It is important to emphasize the fact that, while at 0 and low salinity levels (0, 50 mM) between 7 DA and 40 DA, there were no statistically significant differences; at 100 and 200 mM NaCl, differences appeared for the GR-1-BBKK-24.6196 genotype, indicating the beneficial effect of salinity on the early seedling stage (Table 3, *p* < 0.05). In contrast, genotype GR-1-BBKK-25.6212 showed a gradual increase in seedling length in all four salinity levels over time. This suggests that beyond the seed stage, even at the seedling phase, the combined genotype–salinity effect may influence the growth of *S. europaea*. Particular consideration should be given to the comparison of salinity levels over time as it appears that between 15 and 60 DA there are differences concerning the three lower salinity levels (0, 50, 100 mM), while at 200 mM, no differences are shown (Table 3). This may be attributed to the fact that 200 mM NaCl enhanced the growth rate of *S. europaea* under saline conditions.

## 3. Discussion

This research was based on findings in the literature regarding the life cycle of *S. europaea* as well as the environmental parameters that interfere with the growth and development of plants from the seed to the seedling stage. Based on that, we hypothesized that *S. europaea* seeds need exogenous application of GA_3_ to overcome dormancy. Accordingly, among our hypotheses were that seed germination requires little to no presence of a saline substrate and moderate light intensity for efficient germination. Concerning the seedling stage, we assumed that 200 mM NaCl would enhance seedling growth. Regarding the seed stage, salinity and light intensity accounted for the highest contribution to overall variance, exceeding that of genotype and corresponding interactions. The notable genotypic variations identified in this research correspond with contemporary evidence of considerable intraspecific diversity in halophyte stress responses [22]. The significant genotype × light interaction as studied herein highlights the importance of underlining genetic background when developing propagation protocols. Therefore, this finding emphasizes the need for genotype-specific experimentation in order to establish halophyte cultivation protocols [23]. The application of GA_3_ serves as a hormonal seed priming method, which has been shown to enhance seedling vigor [9]. At the same time, exogenous GA_3_ significantly enhances the seed germination rate by reducing starch and soluble protein levels, increasing soluble sugars, and boosting antioxidant enzyme activities [7]. The literature also indicates that most of the time it exhibits superior efficacy when compared to cold stratification methods [9]. In this study, contrary to our initial hypothesis, no significant differences in the germination parameters measured were observed, except for the Vigor Index I, as analyzed between the control and the highest GA_3_ concentration (0, 500 mg L^−1^). Results contrary to our findings were also found in the study of Gunasekara et al. [8], in which dormancy was observed in *Salicornia brachiata* seeds, with the application of GA_3_ leading to its alleviation and to a subsequent increase in germination rates. This discrepancy between our findings and the existing literature can be attributed to differences in genotypes in terms of seed evolutionary mechanisms or heteromorphism, which may well have gradually altered the morphological structure and genome of the plants [22,24,25]. Although the absence of a significant response to GA_3_ supplementation suggests that the seeds were likely in a non-dormant state at the start of the study, it is highly probable that the primary physiological dormancy was alleviated during the two-month storage at 5 °C as chilling is a well-documented mechanism for dormancy release in halophytes [26]. Consequently, our findings reflect the germination requirements of fully after-ripened or stratified seeds, which are typical of the state that seeds would reach in early spring following winter burial in salt marsh sediments.

Beyond on the genotype and seed priming research, the combination of genotype, light intensity and salinity of the growing medium, along with their three-way and two-way interactions is crucial. In this direction, it will be feasible to improve plant production by maximizing the efficiency of resources and plant material. In this study, all three factors presented significant differences. Specifically, regarding our initial hypothesis, the effects of genotype variation, light intensity levels and the absence of salinity were confirmed. Further analysis indicated a relative reduction in germination percentage and germination speed under saline conditions. The reduction in germination speed index between 0 and 1% NaCl suggests that even small levels of salt stress in early phases of *S. europaea* development partially impedes germination kinetics compared to non-salt-stressed conditions. Ungar et al. [27] also pointed out that optimal seed germination transpires under conditions of diminished salinity, while at the same time highlighting the connection with the ecological framework under which *S. europaea* thrives. Calone et al. [26] came to the same conclusion, as they identified a negative correlation between salt stress and the majority of measured germination parameters in their analysis. This can be attributed to the fact that in coastal areas, which constitute the natural habitat of *S. europaea*, it grows after winter rains, which dilute the salinity concentration in the growing substrate and activate the seed physiological mechanism [27]. Under direct sowing conditions in salinity-degraded soils, Hammed et al. [9] suggest seed priming as the most effective method of alleviating this type of abiotic stress but, at the same time, they highlight the species-specific type of priming focus that should be considered. Concerning the influence of light conditions on seed germination, the absence of light in the growth environment exhibited the most suboptimal performance among the various light intensity conditions examined. A similar conclusion was derived by Lee et al. [28], who highlighted that seed germination of *Salicornia herbacea* was totally enhanced by the combination of seed coat removal, light and a 0 NaCl environment compared to total darkness and various NaCl concentrations. The findings of Khan et al. [17], who indicated a notable decline in the percentage of germination of *Salicornia pacifica* var. *utahensis* when seeds were subjected to germination in total darkness, can be added to the above. Therefore, light constitutes one of the most important factors for the germination of *S. europaea* seeds, and according to our study, it was the most important factor based on the analysis of variance in terms of seed germination speed (Table 2). Regarding the most optimal light intensity level in this study, it appeared to be the highest level tested (80 PAR), which was inconsistent with our hypothesis as we indicated medium light intensity (40 PAR) as the most suitable level for seed enhancement. The significant improvement in germination speed under 80 µmol m^−2^ s^−1^ compared to dark conditions underscores the importance of light availability at the soil surface. Even the relatively low intensity of 40 µmol m^−2^ s^−1^ was sufficient to stimulate a response, suggesting that *S. europaea* is well-adapted to germinate under shaded or turbid conditions common in the early-season salt marsh environment. In line with our findings, Sisay et al. [29] applied a light intensity of 200 µmol m^−2^ s^−1^ during the germination-to-seedling stage in *S. brachiata*, highlighting that the optimum light intensity range for maximizing growth remains inadequately defined (80–200 µmol m^−2^ s^−1^). Regarding the salinity effect on the juvenile phase of *S. europaea*, our results showed a consistent relationship with our initial hypothesis revealing a positive correlation between seedling length and increase in salinity. However, limited insights concerning early seedling establishment exist in the literature and contrary findings were reported by Aghaleh et al. [30], who observed a decline in root length under increasing salinity, and Katel et al. [31], who pointed out that genotypic interference is a crucial factor for seedling establishment and observed a decrease in growth under increasing salinity (200 mM NaCl). The significant stimulation of seedling growth at 200 mM NaCl is a characteristic response of obligate halophytes. Unlike glycophytes, which degrade due to ion toxicity, *S. europaea* aggr. effectively utilizes Na^+^ and Cl^−^ ions for osmotic adjustment [2]. Consequently, this process facilitates turgor-driven cell expansion, which thereafter leads to increased succulence and dilution of accumulated salts to non-toxic levels [4]. Therefore, according to our research findings, the complex mechanism behind the physiology of *S. europaea* seeds is being revealed, compared with the most important parameters for achieving an acceptable germination rate and ensuring optimal seedling growth.

Overall, the observed ontogenetic shift in salinity response—where 1% NaCl inhibited germination speed while 200 mM NaCl stimulated early seedling growth—suggests a transition in physiological priorities. In the germination phase, the primary constraint is likely osmotic, where high external salt concentrations limit the water uptake necessary for embryo protrusion. Conversely, at the seedling stage, the stimulatory effect of 200 mM NaCl suggests that *S. europaea* aggr. has transitioned to the ionic phase of salt tolerance [2,22].

## 4. Materials and Methods

### 4.1. Seed Collection

*Salicornia europaea* aggr. exists in native populations in the coastal saline wetlands/marshes near the city of Thessaloniki in Greece, the Thessaloniki airport (40°32′23.93″ N, 22°58′47.10″ E), Epanomi (40°23′37.71″ N, 22°54′23.95″ E), and Kalochori (40°38′24″ N, 22°52′34″ E). Seeds of *Salicornia europaea* aggr. were collected from two populations, genotype GR-1-BBKK-24.6196 from Epanomi and genotype GR-1-BBKK-25.6212 from Kalochori, with both areas being characterized by regular tidal inundation and high salinity fluctuations. Seeds were collected during peak maturity in November 2024, air-dried, and stored at 5 °C in sealed containers until the experiment (January 2025). This two-month cold storage period may have functioned as a cold stratification phase. The collections of genotypes occurred after receiving a special permit from the Institute of Plant Breeding and Genetic Resources, Hellenic Agricultural Organization–DIMITRA (Permit 25237/1830 of 14 April 2024) issued by the Greek Ministry of Environment and Energy. Following that, each genotype received a distinct IPEN accession number allocated by the Balkan Botanic Garden of Kroussia (BBGK), Institute of Plant Breeding and Genetic Resources (IPBGR), and Hellenic Agricultural Organization–DIMITRA (ELGO–DIMITRA).

### 4.2. GA_3_ Seed Dormancy In Vitro Test of Salicornia europaea aggr.

To evaluate the dormancy status of *S. europaea*, seeds derived from the aforementioned genotypes were subjected to an experimental setup under totally controlled conditions (temperature 22 ± 0.5 °C; relative humidity 60 ± 5%) under LED lights of a 16:8 (light:dark) photoperiod, a 430–690 spectral range and 80 µmol m^−2^ s^−1^ photosynthetically active radiation (PAR) in a growth chamber for 20 days. For both genotypes, seeds were sown in Petri dishes filled with 25 mL of 0.6% agar and divided into three GA_3_ treatments as follows: 0, 250, and 500 mg L^−1^. Prior to that, Petri dishes filled with medium were autoclaved at 121 °C for 20 min while regarding in vitro disinfection, seeds were immersed in Signum^®^ fungicide (BASF Agricultural Solutions, Nunhem, The Netherlands) (0.1 g/100 mL ddH_2_O) for 30 min, followed by incubation in 70% ethanol (Sigma-Aldrich, St. Louis, MO, USA) for 1 min, and then in 3% sodium hypochlorite (Sigma-Aldrich) solution for 30 min, followed by 3 rinses with sterilized ddH_2_O. Regarding the evaluation of germination and growth, parameters such as seed germination (%), germination speed, seedling length, seedling fresh weight, seedling dry weight, Vigor Index I, and Vigor Index II were assessed. The speed of the germination index was calculated according to Maguire [32]:

(1) $$Speed\;of\;germination=\sum \left(\frac{n}{t}\right)$$

where *n* is the number of seeds newly germinating at time *t* and *t* is days after sowing.

Seedling vigor indexes, which represent dimensionless indices, were calculated as follows [33]:

Vigor (Index) I = Germination percentage × Seedling length(2)

Vigor (Index) II = Germination percentage × Seedling dry weight(3)

### 4.3. Genotype × Light Intensity × Salinity In Vitro Effect on Salicornia europaea aggr.

To measure the complex effect of genotype (G) and environment, seeds from genotypes GR-1-BBKK-24.6196 and GR-1-BBKK-25.6212 were studied under experimentation in totally controlled conditions (temperature 22 ± 0.5 °C) under LED lights of a 16:8 (light:dark) photoperiod, a 430–690 spectral range and three light intensity levels (LIs) (dark, 40 and 80 µmol m^−2^ s^−1^ PAR). The light intensities of 40 and 80 µmol m^−2^ s^−1^ were chosen to establish the light requirement threshold for germination, representing low-light and moderate-light conditions. This range is consistent with established protocols for halophytes [34,35]. These light intensities also represent the attenuated irradiance levels reaching the soil surface in coastal salt marshes during the spring. In these habitats, seeds are frequently shaded by plant litter, previous years’ vegetation, or thin layers of sediment. The salinity factor (S) contained two levels (0 and 1% of NaCl) of the medium used, respectively, which were selected based on the previous literature [27] to represent the critical salinity threshold for *S. europaea* germination. Furthermore, this concentration reflects the moderate soil salinity levels typically observed at the collection sites during the early spring rainy season, providing a coherent transition to the 200 mM concentrations used in the subsequent seedling stage. Seeds were sown in Petri dishes filled with 25 mL of 0.6% agar. Regarding the in vitro disinfection, the same protocol as mentioned above was used.

In order to assess the effect of the three-way factors on germination, parameters such as seed germination (%), germination speed, seedling fresh weight, seedling dry weight, Vigor Index I, and Vigor Index II were calculated as described in the previous paragraph.

### 4.4. Salinity and Genotype Effect of In Vitro S. europaea aggr. Seedlings

To test the effect of various NaCl concentrations and genotypes on early growth and establishment of seedlings, 40-day-old seedlings from genotypes GR-1-BBKK-24.6196 and GR-1-BBKK-25.6212 were grown under 0, 100 and 200 mM NaCl. The seedlings were set in test tubes containing Murashige Skoog [36] (MS basal mixture including a vitamin formula, Duchefa B.V., Haarlem, The Netherlands). Each test tube was used as an experimental replicate, and each salinity level contained 20–25 replicates. To assess the effect of the salinity level on early-stage seedling, seedling length was measured at 7, 15, 30, and 60 days after the installation of the experiment (DA). Subsequently, at the end of the experiment (60 DA), the seedling fresh and dry weight were calculated. Concerning dry weight determination, seedlings were placed in an oven at 72 °C for 48 h.

### 4.5. Experimental Design and Statistical Analysis

All experiments were conducted at least twice to ensure reproducibility; no significant deviations were observed between trials. The experimental period was concluded once the germination levels stabilized across treatments (20 days for the seed and 60 days for the seedling experimentation, respectively). Data concerning seed experimentation was analyzed based on a completely randomized design (CRD) with five replications of 25 seeds each, and the means were compared using a post hoc test of honest significance, Tukey’s HSD, and Student’s *t*-test where applicable, at a preset level of α = 0.05. Where homogeneity of variances was not met regarding simple main effects, the Games–Howell test was conducted. Prior to post hoc tests, data residuals were evaluated for normality and primary data for homogeneity of variances using Shapiro–Wilk and Levene’s tests, respectively. To assess the overall effect of each parameter tested on the total variation, partial eta squared effect size index (%) was used as follows: η_p_^2^ = Sum of squares of the effect/(Sum of squares of the effect + Sum of squares of the error).

The statistical software used for all analyses of variances (ANOVAs) was IBM-SPSS v.29, and graphs were drawn using Microsoft Excel.

## 5. Conclusions

*Salicornia europaea* aggr. represents a neglected and underutilized edible species of high importance for sustainable agriculture. Seeds of selected *S. europaea* genotypes exhibited no dormancy during in vitro propagation trials. Regarding the effect of distinct genotypes, PAR, and salinity levels, it was found that the germination of *S. europaea* seeds appeared to be genotype-dependent, and optimal results were achieved under conditions of elevated light intensity (80 µmol m^−2^ s^−1^) and non-saline conditions in the growing medium. The light intensity factor that, in our research findings, exerted the greatest contribution to the variability as measured in the parameters germination speed and Vigor I, especially represents a common experimental factor in halophyte germination within a range of 40–80 µmol m^−2^ s^−1^ [34,35]. Consequently, this combination is proposed as the optimized protocol for in vitro propagation. Regarding the early-stage seedlings grown under different salinity levels, small variations in seedling length were observed, resulting in differences at 60 DA both because of the genotype and NaCl in terms of fresh weight and seedling length. Thus, although salinity seemed to be initially detrimental at the seed stage, at the immediate subsequent stage, it exerted an essential effect on seedling growth. These differences highlight the importance of the genotype and environmental factors effect on the seed and seedling stages. To this end, more focus should be placed on research of the seedling stage, testing various light intensities and salinity levels to unveil the optimal method for successful plant establishment.

## Figures and Tables

**Table 1:** Germination and seedling growth parameters of two genotypes of *Salicornia europaea* aggr. (GR-1-BBKK-24.6196, GR-1-BBKK-25.6212) were evaluated 20 days post-sowing, treated with various gibberellic acid GA_3_ levels (0, 250, 500 mg L^−1^).

Genotype × GA_3_ Level		Germination (%)	Germination Speed Index	Seedling Length (cm)	Seedling Fresh Weight (g)	Seedling Dry Weight (g)	Vigor I	Vigor II
GR-1-BBKK-24.6196	0 mg L^−1^	53.0 ± 32.20 a	5.10 ± 1.21 a	0.71 ± 0.23 a	0.002 ± 0.00 a	0.0007 ± 0.00 a	27.00 ± 11.90 a	0.02 ± 0.02 a
	250 mg L^−1^	58.0 ± 8.32 a	6.30 ± 2.56 a	0.77 ± 0.10 a	0.007 ± 0.00 a	0.0006 ± 0.00 a	43.49 ± 1.74 a	0.03 ± 0.00 a
	500 mg L^−1^	60.0 ± 5.65 a	7.20 ± 0.62 a	0.84 ± 0.23 a	0.005 ± 0.00 a	0.0004 ± 0.00 a	51.92 ± 21.02 a	0.02 ± 0.01 a
GR-1-BBKK-25.6212	0 mg L^−1^	44.8 ± 11.09 a	6.50 ± 2.17 a	0.55 ± 0.12 a	0.003 ± 0.00 a	0.0002 ± 0.00 a	23.18 ± 10.71 a	0.02 ± 0.02 a
	250 mg L^−1^	51.2 ± 3.34 a	6.20 ± 1.17 a	0.54 ± 0.06 a	0.003 ± 0.00 a	0.0004 ± 0.00 a	28.15 ± 1.13 a	0.03 ± 0.00 a
	500 mg L^−1^	60.0 ± 0.00 a	8.02 ± 0.21 a	0.75 ± 0.06 a	0.005 ± 0.00 a	0.0004 ± 0.00 a	45.3 ± 3.99 a	0.02 ± 0.01 a
**Means of Genotype (G)**								
GR-1-BBKK-24.6196		50.6 ± 23.20 a	6.22 ± 1.76 a	0.77 ± 0.18 a	0.004 ± 0.00 a	0.0006 ± 0.00 a	40.81 ± 16.35 a	2.38 ± 1.59 a
GR-1-BBKK-25.6212		50.7 ± 9.00 a	6.80 ± 1.59 a	0.61 ± 0.12 b	0.004 ± 0.00 a	0.0004 ± 0.00 a	32.21 ± 11.74 a	1.97 ± 0.98 a
**Means of GA_3_ (GA)**								
0 mg L^−1^		46.6 ± 22.8 a	5.93 ± 1.87 a	0.63 ± 0.18 a	0.003 ± 0.00 a	0.0005 ± 0.00 a	25.09 ± 10.36 b	1.61 ± 1.72 a
250 mg L^−1^		54.2 ± 6.60 a	6.31 ± 1.77 a	0.65 ± 0.14 a	0.005 ± 0.00 a	0.0006 ± 0.00 a	35.82 ± 8.86 ab	2.95 ± 0.90 a
500 mg L^−1^		52.0 ± 18.61 a	7.56 ± 0.62 a	0.80 ± 0.16 a	0.005 ± 0.00 a	0.0004 ± 0.00 a	48.61 ± 14.01 a	1.96 ± 0.92 a
**Source of variation**		*p*	*p*	*p*	*p*	*p*	*p*	*p*
G × GA		ns	ns	ns	ns	ns	ns	ns
G		ns	ns	*	ns	ns	ns	ns
GA		ns	ns	ns	ns	ns	*	ns

*p*: *p* value; ns: not significant; * significance at *p* < 0.05; different lowercase letters within columns indicate significant differences based on Tukey’s HSD post hoc test.

**Table 2:** Analysis of variance (ANOVA) for the three-way factorial experiment including genotype containing two levels (GR-1-BBKK-24.6196 and GR-1-BBKK-25.6212), light intensity containing three levels (0, 40 and 80 µmol m^−2^ s^−1^ PAR) and salinity containing two levels (0 and 1% NaCl).

		Germination (%)			Germination Speed			Vigor I			Vigor II		
Source	df	SS	*p*	η_p_^2^	SS	*p*	η_p_^2^	SS	*p*	η_p_^2^	SS	*p*	η_p_^2^
Genotype (G)	1	5152	***	50.9	124.7	***	41.6	49,410	***	32.9	0.1	**	28.3
Light Intensity (LI)	2	6754	***	57.6	792.5	***	81.9	159,834	***	61.4	0.5	***	68.5
Salinity (S)	1	7437	***	60.0	561.2	***	76.2	76,127	***	41.1	0.6	***	69.9
G × LI	2	401	ns		14.9	ns		14,143	***	12.6	0.0	ns	
G × S	1	167	ns		7.6	ns		16,030	*	13.7	0.0	ns	
LI × S	2	951	*	16.1	22.0	ns		30,338	**	23.2	0.1	***	35.2
G × LI × S	2	599	ns	10.8	25.4	*	12.7	3251	ns		0.0	ns	
Error	48	4966			175.2			100,347			0.2		
Total	60	184,944			8437.5			2,859,039.2			4.3134		

df, degrees of freedom; SS, sum of squares; *p*, probability; η_p_^2^, partial eta squared effect size index; *** significance at *p* < 0.001; ** significance at *p* < 0.01; * significance at *p* < 0.05 and ns, not significant.

**Table 3:** Seedlings length (cm) of two genotypes of *Salicornia europaea* aggr. treated with various NaCl levels as measured at 7, 15, 40 and 60 days, and fresh weight (F.W.; g) and dry weight (D.W.; g) at 60 days after the installation of the experiment (DA).

Genotype × Salinity Level		7 DA	15 DA	40 DA	60 DA	F.W. (60 DA)	D.W. (60 DA)
GR-1-BBKK-24.6196	0 mM NaCl	1.11 ± 0.52 ab A	1.46 ± 0.73 ab A	1.47 ± 0.98 a A	1.47 ± 0.69 a A	0.008 ± 0.00 a	0.001 ± 0.00 a
	50 mM NaCl	1.16 ± 0.39 a A	1.41 ± 0.61 ab A	1.41 ± 0.52 a A	1.42 ± 0.91 a A	0.012 ± 0.01 a	0.001 ± 0.00 a
	100 mM NaCl	0.96 ± 0.38 ab B	1.28 ± 0.84 ab AB	1.60 ± 1.39 a A	1.62 ± 1.37 a A	0.028 ± 0.03 a	0.003 ± 0.00 a
	200 mM NaCl	1.05 ± 0.43 ab B	1.43 ± 0.61 ab AB	1.93 ± 1.04 a A	1.94 ± 1.18 a A	0.016 ± 0.01 a	0.002 ± 0.00 a
GR-1-BBKK-25.6212	0 mM NaCl	0.74 ± 0.23 b C	1.05 ± 0.44 b BC	1.55 ± 0.74 a AB	1.62 ± 0.94 a A	0.020 ± 0.00 a	0.002 ± 0.00 a
	50 mM NaCl	0.94 ± 0.33 ab C	1.65 ± 0.59 ab BC	2.43 ± 1.69 a AB	2.80 ± 2.04 a A	0.038 ± 0.05 a	0.002 ± 0.00 a
	100 mM NaCl	1.22 ± 0.36 a B	1.78 ± 0.65 a AB	2.30 ± 1.28 a A	2.36 ± 1.38 a A	0.063 ± 0.04 a	0.005 ± 0.00 a
	200 mM NaCl	1.07 ± 0.39 ab B	1.86 ± 0.75 a AB	2.08 ± 1.16 a A	2.63 ± 1.56 a A	0.058 ± 0.02 a	0.005 ± 0.00 a
**Means for genotypes**							
GR-1-BBKK-24.6196		1.07 ± 0.43 a B	1.4 ± 0.70 a AB	1.59 ± 1.03 b A	1.60 ± 1.06 b A	0.016 ± 0.02 b	0.009 ± 0.00 a
GR-1-BBKK-25.6212		0.99 ± 0.37 a C	1.58 ± 0.68 a B	2.11 ± 1.28 a A	2.33 ± 1.58 a A	0.045 ± 0.03 a	0.004 ± 0.00 a
**Means for salinity level**							
0 mM NaCl		0.93 ± 0.44 a B	1.27 ± 0.64 a AB	1.50 ± 0.87 a A	1.52 ± 0.82 b A	0.014 ± 0.01 a	0.001 ± 0.00 a
50 mM NaCl		1.05 ± 0.37 a B	1.53 ± 0.60 a AB	1.93 ± 1.35 a A	2.13 ± 1.72 ab A	0.025 ± 0.03 a	0.002 ± 0.00 a
100 mM NaCl		1.09 ± 0.38 a B	1.54 ± 0.78 a AB	1.96 ± 1.36 a A	2.00 ± 1.41 ab A	0.046 ± 0.04 a	0.004 ± 0.00 a
200 mM NaCl		1.06 ± 0.41 a C	1.65 ± 0.71 a BC	2.03 ± 1.09 a AB	2.28 ± 1.41 a A	0.037 ± 0.03 a	0.003 ± 0.00 a
**Source of variation**		*p*	*p*	*p*	*p*	*p*	*p*
G × S		**	**	ns	ns	ns	ns
G		ns	ns	**	**	*	ns
S		ns	ns	ns	**	ns	ns

*p*: *p* value; ns: not significant; ** significance at *p* < 0.01; * significance at *p* < 0.05; different lowercase letters within each column section and uppercase letters between columns indicate significant differences based on Tukey’s HSD and Games–Howell (where applicable in simple main effects) post hoc tests.

## Data Availability

Data is contained within the article. The original contributions presented in the study are included in the article; further inquiries can be directed to the corresponding author.
